# Tetanus Vaccination Status and Vaccine Hesitancy in Amateur Basketball Players (Italy, 2020)

**DOI:** 10.3390/vaccines10010131

**Published:** 2022-01-17

**Authors:** Matteo Riccò, Simona Peruzzi

**Affiliations:** 1 Dipartimento di Sanità Pubblica, Servizio di Prevenzione e Sicurezza Negli Ambienti di Lavoro (SPSAL), AUSL—IRCCS di Reggio Emilia, Via Amendola n.2, I-42022 Reggio Emilia, Italy; 2 Laboratorio Analisi Chimico Cliniche e Microbiologiche, Ospedale Civile di Guastalla, AUSL—IRCCS di Reggio Emilia, I-42016 Guastalla, Italy; simona.peruzzi@ausl.re.it

**Keywords:** tetanus, knowledge, attitudes, practices, risk perception, immunization, health knowledge, sport

## Abstract

Basketball is among the third most popular team sport in Italy. Albeit not usually perceived as being at high risk for tetanus, the Italian legal framework (Law No. 292 of 1963; Presidential Decree 1301/1965) requires tetanus vaccination (TeV) even for amateur practice. Even though some previous reports have suggested a relatively low adherence towards vaccination practice among basketball player, corresponding knowledge, attitudes and practices towards TeV remain largely unknown. Our study specifically investigated such topics in a total of 270 amateur basketball players participating into an internet-based survey by completing a structured questionnaire. Of them, 73.0% had a proper vaccination status, but a third of respondents (33.3%) exhibited some degree of vaccine hesitancy. The average understanding of TeV and tetanus (79.8% with a potential range 0–100) as well as the risk perception for natural infection (63.9% ± 26.6) were quite good. Even though unmotivated fears towards TeV were more scarcely reported (14.0% ± 15.4), they still represented the main reasons for having missed vaccination shots (63.0%). Knowledge status and risk perception for natural infection and TeV were well correlated (R = 0.22 and R = −0.64, respectively). Appropriate TeV status was more likely in respondents not exhibiting vaccine hesitancy (Odds Ratio (OR) 0.114, 95% Confidence Interval (95%CI) 0.059–0.225). In turn, vaccine hesitancy was more frequently reported among individuals of male gender (OR 3.148, 95%CI 1.072–9.244), while better formal education (OR 0.065, 95%CI 0.013–0.319) and working in healthcare settings (OR 0.042, 95%CI 0.007–0.265) were characterized as negative effectors. Vaccinations in athletes represent an often overlooked issue, with a considerable lack of available evidence. The results stress the opportunity for appropriate TeV screening programs among amateur athletes and the potential relevance of interventions aimed at raising the perceived significance of TeV in order to cope with a significant share of vaccine-hesitant athletes.

## 1. Introduction

Tetanus is a severe and potentially deadly disease caused by a neurotoxin produced by the spore-forming anaerobic bacteria *Clostridium tetani* [1,2]. Improving tetanus vaccination rates by all available means is of critical importance for public health [1,3,4], particularly in Italy. Since the early 1960s, a specific legal framework (Law No. 292/63) identified tetanus vaccination (TeV) as compulsory for all people born after 1968 as well as for workers engaged in activities considered to be at risk for interaction with tetanus toxin (e.g., construction, farming, waste collection and animal husbandry) [5,6,7,8]. However, such requirements are largely disregarded. According to available figures, up to 20% of all Italian population is reportedly susceptible to tetanus because of inadequate boost doses, and serologic surveys have pointed out that up to 40% of Italian population has inadequate protection [4,6,7,9,10]. Not coincidentally, since 2006, Italy has consistently reported the highest number of European cases [4,5,6,7,9,10,11]. Despite a significant drop in notification rates from 0.12 cases/100,000 inhabitants in 2013 to 0.04 cases/100,000 inhabitants in 2017, 65% of all cases reported in EU/EEA still occur in Italy [6,12]. In order to cope with such unsatisfying vaccination rates as well as with the re-emergence of anti-vaccination movements [13,14,15], the National Vaccination Prevention Plan 2017–2019 (NVPP) has strongly encouraged the active decennial offer of TeV either as diphtheria toxoid (Td) formulate or tetanus toxoid, reduced diphtheria toxoid and acellular pertussis (Tdap) in all adults, exploiting all interaction with subjects at higher risk for inappropriate vaccination status, also including periodic sport medicine checkups [13,15,16].

Some previous reports from United States have pointed out that both professional and amateur (including collegiate) athletes may be affected by inadequate vaccination rates and significant vaccine hesitancy, particularly among basketball players (BPs), and the causes still remain unclear [17,18]. For example, in a sample including 98 professional athletes, with 36 of them being professional BPs, the risks for inadequate immunity to varicella and rubella were 4 and 6 times higher, respectively, than in a general age-matched population [18]. More recently, the vaccination campaigns for SARS-CoV-2 also have faced substantial resistance among professional BPs [19,20].

According to the Italian National Institute of Statistics (ISTAT), around 2 million people practice or have actively practiced basketball during their lifetime (6% of the total population) [21]. With around 800,000 active professional and amateur athletes, basketball ranks as the third most popular team sport [22]. Even though basketball is at relatively low risk for penetrating injuries [16,23,24], with subsequent potential contamination by spores of *C. tetani*, BPs share the common requirement for all Italian athletes affiliated with the National Olympic Committee (CONI, in Italian) to be vaccinated against tetanus [16]. To date, accurate estimates of the actual TeV rates among BPs, and particularly among amateur BPs, are not available. In this regard, it should be stressed that the Presidential Decree 1301/65 establishes that athletes who do not have vaccine coverage or who have not received TeV booster doses cannot be registered with or must be canceled by the federation to which they belong [16]. Therefore, while the adherence of professional athletes to official requirements on vaccines, including TeV, substantially represents a legal framework for their contract, amateur BPs may represent a relatively large group of sporters characterized by relatively low risk perception for tetanus and a potentially low acceptance of vaccine recommendations. As a consequence, the assessment of their *knowledge* (i.e., the awareness of official recommendations), *attitudes* (i.e., propensity towards vaccinations) and *practices* (i.e., actual uptake of vaccination) (collectively known as KAP) on TeV has the potential to improve our understanding of this specific subgroup of athletes, eventually disclosing whether they represent an unexpected “*core group*” for TeV hesitancy.

Our primary objective was therefore to investigate adherence to TeV schedule in a sample of amateur BPs. Our secondary objective was then to investigate their respective KAP on TeV, specifically focusing on vaccine hesitancy and its main drivers.

## 2. Materials and Methods

### 2.1. Study Design

A cross-sectional questionnaire-based study was performed between 1 December 2019 and 31 January 2020, involving participating in 7 different private Facebook group pages and 5 discussion groups on basketball. A total of approximately 400,000 unique members were eventually reached, but no information could be obtained regarding cross-inscriptions, not even how many of these members were actively using the parent platform at the time of the survey. Similarly, no information about the actual share of active BP among the group members was available at that time. As no previous studies on KAP towards TeV have been previously performed among BP in Italy but previous studies on KAP towards TeV have identified a vaccine hesitancy peaking to 20% in certain population groups [12,13,14], assuming a Type I error of 5% (0.05) and a power of 95%, the minimum sample size was calculated as follows:

N = 1.962 × 0.8 × (1 − 0.8)/0.05^2^ = 3.8416 × 0.8 × 0.2/0.0025 = 246 (1)

To post the study invitation, the chief researcher contacted the administrators, requesting preventive authorization to post the link to the questionnaire, including a short description of the aims of the survey. Users who clicked on the invitation texts were provided with the full study information, an opportunity to give their informed consent and a web link to the survey (Google Forms; Google LLC; Menlo Park, California, CA, USA). The survey was conducted in Italian.

To be included in the sample, the participant had to be living in Italy, aged 18 years or older, and an amateur BP. To be considered an amateur BP, the participant had to participate in a formally registered basketball team from any Italian division. Professional and semi-professional players (i.e., individuals who were paid or received a wage for their participation in the basketball team) as well as individuals not residing in Italy at the time of the survey were excluded. If a potential participant was found not to match the inclusion criteria, the survey closed down. The survey was anonymous, and no personal data, such as name, IP address, email address or personal information unnecessary to the survey, were requested, saved or tracked. No monetary or other compensation was offered to the participants.

### 2.2. Questionnaire

The test–retest reliability of the questionnaire was preventively assessed through a survey on 20 amateur players of another team sport (i.e., volleyball) completing the questionnaire at two different points in time. A correlation coefficient was calculated to compare the two sets of responses: items having a coefficient >0.80 were interpreted as consistent and were therefore included in the questionnaire used in this survey. All questions were self-reported and not externally validated. An English translation of the questionnaire is available as Appendix A at the end of the present paper. The final questionnaire included the following sections:

#### 2.2.1. Individual Characteristics

Age, sex, education level, whether they had any migration background, whether they lived with children (i.e., individuals < 12 year-old) and whether they had any occupational background in healthcare settings were assessed.

#### 2.2.2. Potential Interactions with Tetanus and TeV

Whether they had any previous interaction with a tetanus case and whether their main occupation or hobbies required vaccination against tetanus according to the National Law 292/63 (see Appendix B for a direct English translation) were assessed. Participants were then assessed regarding their TeV status. According to the Italian NVPP 2017–2019, a complete set of TeV includes 3 initial doses that, in newborns, are performed at the 3rd, 5th and 11th months of age, followed by a further shot between age 5 to 6 years, followed by a fifth dose between ages 12 to 18 years, all of them delivered within combined formulates [13,15,16]. According to available guidelines from the WHO [25], a complete schedule for adults with no previous immunization includes 3 initial doses (at T0, T + 4 weeks and T + 6 months), a further dose at T + 1 year and a fifth dose in the following year. In the present study, participants were asked if they were able to recall a basic schedule. Being able to recall a full basic schedule, with their last shot in the previous 10 years, irrespective of its settings (either as a last shot from a basic schedule or as a booster) identified the TeV status as being complete. The setting of their last vaccination shot was also recalled.

#### 2.2.3. Interactions with a Physician

Participants were asked whether they had discussed TeV with a general practitioner (GP), a sports physician (SP) or their occupational physicians (OP).

#### 2.2.4. Risk Perception

Participants were initially asked to rate the perceived severity (C^T^) and the perceived frequency (I^T^) of tetanus in Italy by means of a fully labeled 5-point Likert scale. The available options ranged from “not significant” (i.e., “of no significant concern in daily practice”, score 1) to “very significant” (i.e., “of very high concern in daily practice”, score 5). Similarly, participants were then asked about the perceived severity (C^V^) and frequency (I^V^) of the side effects of TeV. As perceived risk has been defined as a function of the perceived probability of an event and its expected consequences [26,27,28], two distinctive risk perception scores (RPS) were eventually calculated as follows and reported as a percent value:

RPS-T = I^T^ × C^T^ (2)

RPS-V = IV × CV(3)

#### 2.2.5. Knowledge Test

Participants received a knowledge test including a set of 13 true–false statements on tetanus vaccination that were previously validated in KAP studies on vaccine hesitancy and particularly on TeV KAP in Italian subjects [12,13,23,24]. A summary score (general knowledge score, GKS) was eventually calculated as follows: when the participants answered correctly, +1 was added to a sum score, whereas a wrong indication or a missing/“do not know” answer added 0 to the sum score. GKS was dichotomized by median value in higher vs. lower knowledge status.

#### 2.2.6. Attitudes and Practices

Vaccine hesitancy has been defined as a “delay in acceptance or refusal of vaccine despite availability of vaccination services” [28]. Focusing on a key aspect of the acceptance of an intervention, in which the need may be improperly perceived by a substantially adult individual, we tentatively characterized vaccine hesitancy towards TeV by means of the Transtheoretical Model (TTM), an integrative, biopsychosocial model to conceptualize the process of intentional behavior change [29,30]. The heart of this model is acknowledging that, while modifying a certain behavior (e.g., receiving or not receiving TeV), a person moves through a discrete set of constructs, in a cyclic series of “stages” of readiness (i.e., precontemplation, “not ready/not interested towards vaccination”; contemplation, “preparing to be ready to be vaccinated”; preparation “being ready to be vaccinated”; action, “being vaccinated”; and maintenance, “monitoring vaccination status”), for which the theoretical bases are decisional balance, self-efficacy and processes of change [29,30]. As vaccine hesitancy has been described as a continuum between complete acceptance and complete refusal [31], the application of a dynamic model such as the TTM may be particularly useful to properly characterize KAP of study participants, allowing for the implementation of accurate and tailored interventions, able to assist in the progressive shift in individuals through the stages of changes [32,33].

A series of 10 statements about TeV, ranging from “I am not interested in obtaining TeV” (i.e., precontemplation) to “I have completed the vaccination schedule; I have noted the need for further shots” (i.e., termination) were therefore presented to the study participants, asking them to mark the statement more akin to their attitude towards TeV, allowing for a rating according to the TTM stages. In the present study, people in the precontemplation (i.e., people who do not intend to take action, who are unaware that their behavior is problematic or may produce negative consequences, who underestimate the pros of changing behavior and who place too much emphasis on the cons of changing behavior) and contemplation (i.e., people intend to start the healthy behavior in the foreseeable future as they acknowledge that their behavior may be problematic) stages were assimilated into the vaccine hesitancy category, and the attitudes of the participants were therefore dichotomized as somewhat hesitant vs. somewhat favorable to TeV. The cut-off between the preparation and contemplation stages (and therefore in hesitation vs. having favorable attitudes toward TeV) was arbitrarily identified as being interested in obtaining TeV in the 30 days since completion of the questionnaire. This choice was based on the requirements of the Italian National Health Services at the time of the study, as individuals interested in becoming vaccinated usually had to personally go to vaccination centers, with obvious conflicting schedules with work and other personal requirements [10].

### 2.3. Data Analysis

Continuous variables were initially tested for normal distribution (D’Agostino and Pearson omnibus normality test), where the corresponding *p*-value was <0.10; “normal” distribution was assumed as rejected; and bivariate correlations between continuous variables were compared using Spearman’s rank test. On the other hand, bivariate correlation between variables passing the normality check (D’Agostino and Pearson *p*-value ≥ 0.10) was assessed by calculating the Pearson’s correlation. Categorical variables were reported as percentages, and their distributions with respect of the outcome variables were initially analyzed using a chi-squared test. Two outcome variables were specifically assessed: reporting an appropriate vaccination status for TeV; showing any vaccine hesitancy.

All categorical variables that, at univariate analysis, were associated with the aforementioned statuses with a *p*-value < 0.05 were included as explanatory variables in a stepwise binary logistic regression analysis model of having an appropriate TeV status and exhibiting any vaccine hesitancy. Adjusted odds ratios (adjOR) and their respective 95% confidence intervals (95%CI) were calculated accordingly. All statistical analyses were performed by means of IBM SPSS Statistics 25.0 for Macintosh (IBM Corp. Armonk, NY, USA).

### 2.4. Ethical Considerations

Before giving their consent to participate in the survey, participants were briefed that all information would be gathered anonymously and handled confidentially. Participation was voluntary, and the questionnaire was collected only from subjects who had expressed consent for study participation. Identification of individual participants by means of the presented material was impaired by the lack of personal data such as the community of residence, the precise occupational setting, etc. Due to the anonymous, observational design and the lack of clinical data about patients, as the study did not configure itself as a clinical trial, a preliminary evaluation by an Ethical Committee was not required, according to the Italian law (Gazzetta Ufficiale no. 76, dated 31 March 2008).

## 3. Results

### 3.1. Descriptive Analysis: General Characteristics of the Sample

As shown in Table 1, a total of 270 participants eventually completed the online questionnaire (0.06% of the targeted population). Of the respondents, 36 (i.e., 13.3%) were aged 50 years or more (mean age: 36.9 years ± 12.0); 57.4% were females, and 42.6% were males. A total of 31.5% reportedly lived with other younger than 12 years, and only five individuals (1.9%) had a migration background. Overall, the majority of respondents (59.6%) reported a university-level of educational achievement, and around a third of the sample (34.4%) worked in healthcare settings.

### 3.2. Previous and Potential Interactions with TeV

Overall, 13.0% of respondents reportedly worked in occupational settings where TeV is statutorily required. A total of 123 participants (45.6%) reported any hobby/leisure activities potentially associated with exposure to the spores of tetanus. Eventually, 19 individuals (7.0%) had a previous interaction with tetanus cases during their lifetime (i.e., tetanus occurring among subjects from their families, among relatives or friends, or simply in individuals they personally knew).

### 3.3. General Knowledge Test

**Figure 1:**
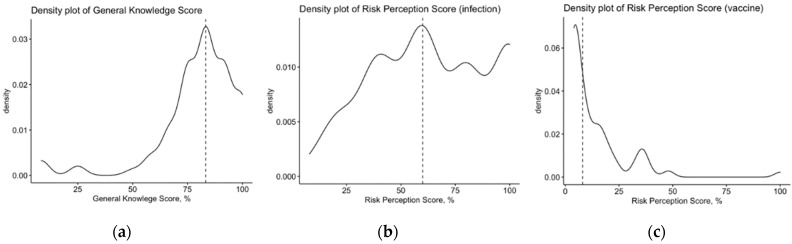
Density plots for the general knowledge score (**a**), and the risk perception scores regarding natural infection (**b**) and vaccines (**c**). All cumulative scores are reported as percent values. Dotted lines represent median values.

After percent normalization, mean GKS accounted for 79.8% ± 16.6 (median 83.4%). Despite a relatively high average score, the distribution was extensively skewed, as confirmed by D’Agostino–Pearson normality test (*p* < 0.001) (Figure 1a). However, the internal consistency coefficient amounted to Cronbach’s alpha = 0.745, suggesting acceptable reliability of the questionnaire.

The details of the knowledge test are shown in Table 2. Briefly, the main uncertainties were associated with some specificities of tetanus and TeV. For instance, 61.9% of respondents acknowledged the specific legal requirements of TeV and 30.4% of them were aware that TeV is required for sport activities, even for basketball. Furthermore, while the majority of participants had some knowledge that tetanus may be acquired through injuries contaminated by earth and dusts (90.4%), only one third of them correctly associated tetanus with improperly managed burns (34.4%).

### 3.4. Risk Perception

The majority of respondents characterizes tetanus as a disease of significant severity and quite common occurrence. In fact, 91.1% of them acknowledged tetanus syndrome severity as severe/very severe, with 59.6% reporting the disorder as common or even very common. A correspondent RPS-T equal to 63.9% ± 26.6 (D’Agostino–Pearson *p*-value < 0.001; Figure 1b) was then calculated (median = 60.0%). Focusing on vaccine-related events, only 6.3% of respondents acknowledged side effects as being common/very common, while 13 respondents (1.1%) identified severe or even very severe side effects as possibly associated with the vaccine, with a cumulative RPS-V equal to 14.0% ± 15.4 (median = 8.0%).

### 3.5. Attitudes and Practices towards TeV

**Figure 2:**
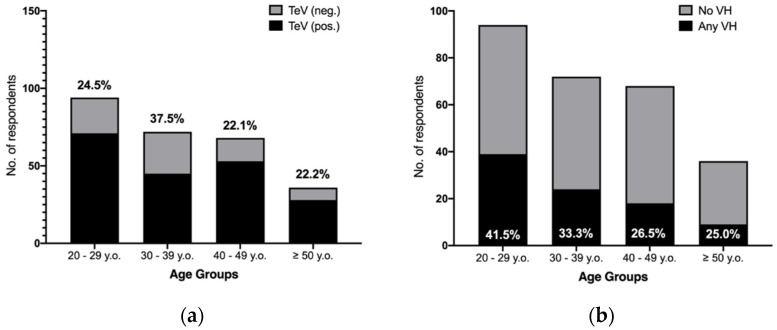
Frequency of appropriate tetanus vaccination (TeV) status (**a**) and vaccine hesitancy (VH) (**b**) by age groups in 270 amateur basketball players participating into the survey.

Overall, 197 out of 270 participants presented an appropriate TeV status (79.8%). The share of properly vaccinated individuals were 24.5% in subjects 20–29 y.o., 22.1% among individuals aged 40 to 49, and 22.2% for older age groups (i.e., > 50 years) (Figure 2a).

According to the respondents, vaccination shots were mostly performed by the local health unit (No. 128, 47.4% of total respondents), followed by a GP (7.4%), by an OP (3.3%) and by professionals of emergency departments as a consequence of an injury (3.0%), while 32 participants were either unable or unwilling to recall the circumstances of the last vaccination shot (11.9%). The healthcare professional most frequently checking TeV status was a GP (27.0%), followed by a competent OP (25.6%) and an SP (17.4%). However, only 37.0% of the respondents were assessed for their TeV status anytime in the past, or conversely, the majority of respondents were never checked by a GP, an SP or an OP.

As shown in Table 3, some degrees of vaccine hesitancy were reported by 33.3% of total respondents, including 8 participants (3.0%) not interested in obtaining TeV, 31 individuals (11.5%) uninterested in being vaccinated anytime sooner than 6 months from the completion of the questionnaire and 2.6% of respondents still uncertain about the eventual acceptance of TeV. Moreover, 1.5% of participants were considering discussing the vaccine with a health professional and 14.8% were reportedly interested in receiving the vaccine in the following 6 months, even though no actual intervention (e.g., scheduling a meeting with a physician, planning a discussion with an healthcare provider, etc.) had been organized.

On the contrary, the majority of respondents showed a somewhat favorable attitude towards TeV (66.7%). This subgroup included individuals in the preparation (i.e., being interested in obtaining TeV in the next 30 days (3.3%), having consciously received the vaccination shot (2.2%), and having consciously received the vaccination but no plans for further shots (4.4%)), maintenance (having either noted need for further shots (28.1%) or made appointments for further shots (5.9%)), and termination (i.e., vaccination schedule consciously completed and noted the need for further shots, 22.6%) phases.

Interestingly, vaccine hesitancy showed a clear age-dependent trend, as the corresponding prevalence was considerably higher among younger participants (41.5% in individuals aged < 30 y.o.) than in older age groups (33.3% among 30–39 y.o, 26.5% among 40 to 49 y.o., and 25.0% in individuals 50 y.o. or older; chi squared test *p*-value < 0.001) (Figure 2b).

The main reasons for becoming vaccinated or for conversely refusing TeV are summarized in Table 4. Briefly, the majority of respondents identified their main motivation for being vaccinated as avoiding tetanus syndrome (111 out of 197; 56.3%). Residual triggers were either represented by a contingent event (i.e., previous penetrating injury treated at the emergency department; 17.1%) or by requirements from personal activities (13.2%) or their main occupation, either following a statutory mandate (5.6%) or specific requirements from the employer (1.5%). On the contrary, recommendations by medical professionals, such as their GP (4.6%), OP (1.5%) or SP (1.5%), played more marginal roles.

Focusing on the barriers towards the acceptance of TeV shots, the majority of respondents reported a fear of side effects (63.0%), followed by a lack of confidence in TeV (16.5%) and doubts about the economic interests of vaccine producers (9.6%). Only 5.5% reported their preference on alterative measures, while four participants forgot to obtain their vaccination shot (5.5%).

### 3.6. Univariate Analysis

**Figure 3:**
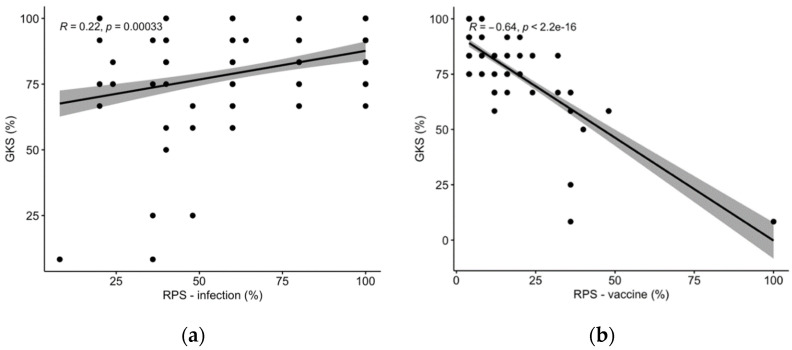
Scatter plots comparing the general knowledge score (GKS) vs. the risk perception score (RPS) for infection (**a**) and vaccine (**b**).

In correlation analyses, GKS and RPS-T were positively correlated (RPS-T, Spearman’s rank correlation test R = 0.22, *p* < 0.001, Figure 3a), while a negative correlation was identified between GKS and RPS-V (RPS-V R = −0.64, *p* < 0.001, Figure 3b), i.e., the better the understanding of tetanus and TeV, the higher the perceived risk of a natural infection and the lower the reception of TeV).

The association between cumulative scores (GKS, RPS-V and RPS-T) and the outcome variables represented by appropriate TeV status (i.e., three separate doses and at least one vaccination shot in the 10 years preceding the study) and by vaccine hesitancy (i.e., being in the precontemplative or contemplative stages according to TTM) is reported in Table 5. Briefly, a better GKS was associated with a lower occurrence of vaccine hesitancy (22.2% vs. 41.1%, *p* = 0.003), while no substantial association with vaccination rate was identified (*p* = 0.157). On the contrary, an appropriate TeV status was more frequently reported among individual scoring a higher RPS-T (46.7% vs. 30.1%, *p* = 0.021) and a lower RPS-V (57.5% vs. 37.6%, *p* = 0.005). Conversely, both scores had no substantial correlation with vaccine hesitancy.

Regarding the personal characteristics of the participants, individuals of the male gender less frequently reported an appropriated TeV status (36.5% in individuals who are properly vaccinated vs. 58.9% among individuals without an appropriate TeV status; *p* = 0.002), and 57.8% of vaccine-hesitant individuals were male (*p* = 0.001). While age, migration background, characteristics of the household and even previous interactions with tetanus had no influences on vaccination status and vaccine hesitancy, higher formal education had an influence. In fact, participants 67.5% of properly vaccinated respondents reported an educational attainment of university level or even higher compared with 38.5% of other respondents (*p* < 0.001). Similarly, highly educated individuals represented 44.4% of vaccine-hesitant respondents compared with 67.2% of participants without signs of vaccine hesitancy (*p* < 0.001).

An assessment of occupational background was associated with mixed results. On the one hand, work-related requirements were more often reported among non-properly vaccinated respondents than among individuals with a proper TeV status (10.2% vs. 20.2%, *p* = 0.040). On the other hand, having a background in healthcare settings resulted in less vaccine hesitancy (22.2% vs. 40.6% for individuals from other occupational sectors, *p* = 0.004).

Similarly, the role of medical professionals was also somewhat inconsistent. While a previous check conducted by an OP or a GP of TeV status was more frequently reported by individuals who were not vaccine-hesitant than by vaccine-hesitant individuals (48.7% vs. 25.9%, *p* = 0.009; 33.9% vs. 13.3%, *p* = 0.001, respectively), being checked by an SP was associated with both vaccine hesitancy (27.8% vs. 12.2%, *p* = 0.002) and improper TeV status (32.9% vs. 11.7%, *p* < 0.001).

Finally, vaccine hesitancy was negatively associated with TeV status (18.8% of properly vaccinated were also vaccine-hesitant vs. 72.6% of improperly vaccinated respondents, *p* < 0.001). In turn, an appropriate TeV status was more frequently reported among non-vaccine-hesitant respondents (88.9%) than among vaccine-hesitant respondents (41.1%; *p* < 0.001).

### 3.7. Multivariable Analysis

In the multivariable analysis (Table 6), potential effectors of outcome variables were assessed through two distinctive models that included the following explanatory variables (all of them were associated with *p* < 0.05 at univariate analysis):

Both outcome variables (i.e., TeV status and any hesitancy): male gender, formal education, previous check of TeV by an SP;

TeV status: the referral of occupational requirements for TeV, higher RSP (for both natural infection and TeV) and any vaccine hesitancy were assessed as explanatory variables for TeV status only;

Reporting any vaccine hesitancy: working in healthcare settings, TeV checked by healthcare providers (including GP and OP), higher GKS and appropriate TeV.

Eventually, only vaccine hesitancy was identified as a possible effector for TeV status (adjOR 0.115, 95%CI 0.059 to 0.225). On the other hand, vaccine hesitancy was positively associated with male gender (adjOR 3.148, 95%CI 1.072 to 9.244) and negatively associated with a series of factors including higher educational level (aOR 0.065, 95%CI 0.013 to 0.319), working in healthcare settings (adjOR 0.042, 95%CI 0.007 to 0.265) and reporting an appropriate TeV status (adjOR 0.030, 95%CI 0.006 to 0.151). The negatively associated one therefore represent negative predictors of vaccine hesitancy.

## 4. Discussion

In our cross-sectional, web-based survey on a select subgroup of the general population (i.e., amateur BPs), we identified a relatively high immunization rate for tetanus (73.0%), exceeding acknowledged Italian vaccination rates (20–40%) [4,6,7,9,10]. Moreover, the participants’ understanding of tetanus and TeV (average GKS 79.8% ± 16.6) and their risk perception for a natural tetanus infection (average RPS-T 63.9% ± 26.6) were quite higher than expected from some previous occupational studies [7,9,10]. Even though fears towards TeV were scarcely reported (average RPS-V 14.0% ± 15.4), they still represented the main reasons for having missed vaccination shots (63.0%).

In the present study, vaccine hesitancy was assessed by means of the TTM, and its design may be particularly useful in tailoring specific interventions for the different stages of hesitancy [30,34]. In fact, the application of TTM highlighted that, around 14.5% of participants may be considered not only vaccine-hesitant but also, more properly, uninterested in receiving TeV, representing “vaccine-resistant” individuals. Conversely, the majority of individuals scoring some degree of vaccine hesitancy were in the “contemplative” stage of change, i.e., ambivalent towards changing their behavior, and more precisely, accepting TeV [29,35]. Such individuals may be properly targeted by specific interventions, as they have some understanding that their behavior may be somewhat problematic and are keen toward assessing the pros and cons of changing their attitudes and practices.

The multivariable analysis showed that the main effectors of vaccine hesitancy were represented by educational attainment and working in healthcare settings, both associated with a better acceptance of TeV, and by being of the male gender, more frequently reported among vaccine hesitant respondents. In turn, vaccine hesitancy was the sole effector of TeV status, stressing how interventions that target hesitancy may effectively improve vaccination rates, also among athletes.

When discussing the prevalence of vaccine hesitancy and the vaccination rates among athletes, several key aspects must be kept in mind. First and foremost, as previously addressed by Tafuri et al., the theme of vaccinations among athletes is not largely studied [36]. Second, the relatively scarce evidence that has been made available is generally focused on professional athletes, while our study specifically addressed KAP from amateur athletes [17,23,37]. Not only are professional athletes routinely screened by an SP for a series of communicable disorders, paying particular attention to the higher risk for severe infections in professional athletes compared with the general population, but also their teams can use economic leverage on hesitant players [18]. For example: recently, some professional basketball players accepted SARS-CoV-2 immunization after initial and considerable resistance when their parent teams were allowed to withhold their salary in cases of games missed due to vaccination mandates (in several states, such as California, unvaccinated individuals are not allowed to participate in mass gatherings and such requirement also extend to athletes) [19,20]. Even though a periodic assessment by an SP is also required for Italian amateur BPs and despite the mandatory status of TeV for all individuals affiliated with the CONI, the current Italian legal framework is quite complicated [16,38,39]. Although the current standards for health surveillance in athletes have been issued nearly two decades after the original requirements for TeV in sport practice [8,40], TeV status was not included among the formal requirements for sports fitness judgement by an SP [39,40]. As a consequence, in analogy with the occupational mandate, a considerable share of athletes may be improperly immunized but still acknowledged as fit for athletic competitions. Not coincidentally, some Italian sports federations (e.g., rugby) and regional governments (e.g., Tuscany Regional Law no. 35, 2003) have issued specific regional laws aiming to reinforce the original requirements, an intervention that, again, mirrors that from occupational immunizations, at least in healthcare settings [39,41]. Third, the majority of available studies on sport teams have been performed in the USA, where a significant share of professional and collegiate athletes comes from ethnic minorities, which have a long history of mistrust towards federal health-related interventions, including vaccinations [17,23,36,38,42,43,44]. Fourth, the evidence collected to date on vaccine acceptance and immunization rates in athletes was gathered on immunizations such as seasonal influenza vaccine, measles or even varicella, i.e., disorders with a significant direct inter-human spread [17,18,36,38,42,43,44]. As tetanus is a non-communicable infectious disease [1,2], the comparisons may be therefore quite misleading.

This is particularly interesting, as the acceptance of all interventions may be understood as a sort of trade-off between what the intervention offers, even from the sole perspective of the recipients, and what the targeted individuals feel as a personal need [26,45,46]. On the one hand, TeV only represents a marginal preventive intervention for a sport with the characteristics of basketball: with physical contact strictly forbidden by rules, without a considerable risk for penetrating injuries, and being a sport played on cement or synthetic playgrounds that have a very limited degree of contamination by spores of *C. tetanii* [6,7,14], the risk for developing tetanus as a consequence of basketball practice is substantially scant. With individuals failing to prioritize TeV shots, vaccination schedules have to be made consistent with personal and training schedules. In other words, it is very unlikely that an amateur basketball player may perceive TeV as a need to be rapidly fulfilled. On the other hand, there is some evidence from Italian studies on TeV in occupational settings that a significant share of individuals, even among professionals who require TeV, may simply leave TeV behind other personal tasks and requirements, advocating the “lack of time” for booking and performing the required shots [12,13,14]. Not coincidentally, even though only a limited share of participants simply “forgot” their vaccination shots (5.5%), up to 17.1% of individuals with an appropriate TeV status reported their last vaccination shots as performed at the emergency department following a penetrating injury. Such a statement suggests that they simply forgot the periodic shot in the previous years, eventually inflating the share of participants affected by the low and improper prioritization of TeV [1,2,47].

Another element to be considered is the amateur status of the study participants: as participants do not receive money for taking part in their basketball teams, all have personal occupations, where TeV may be required by their legal framework [3,7,9,10,48,49]. In fact, 13.0% of them reported the need for TeV as an occupational requirement, with a far larger share of individuals claiming personal hobbies that in turn result in a mandatory status for TeV (45.6%). Individuals with personal backgrounds where a TeV mandate does exist may exhibit increased vaccination rates and better attitudes towards TeV (i.e., less vaccine hesitancy) because of the increased familiarity with the vaccine [48,49]. In fact, the data collected dismissed such a hypothesis. On the one hand, less than 20% of respondents with an up-to-date TeV status advocated for TeV being a requirement either in occupational settings or for personal activities as the main motivator for having been vaccinated. On the other hand, previous interactions with an OP—the medical professional responsible for medical surveillance and health promotion on the workplaces—were substantially unrelated with both vaccination status and reporting vaccine hesitancy [26,50].

In this regard, it is somewhat interesting to stress that the main effector for vaccine hesitancy, but not for vaccination status, was identified in having an occupational background in healthcare settings. Healthcare workers have a mixed reputation in terms of attitudes towards vaccines and immunizations [51,52,53,54], but it is quite reasonable that individuals with at least a basic understanding of the pros and cons of vaccinations may also exhibit less vaccine hesitancy, which in turn, results in an appropriate vaccination status.

Similarly, education status has been often associated with mixed attitudes towards acceptance of vaccinations: particularly in the general population, evidence may be retrieved pointing towards a better and surprisingly low acceptance among more educated individuals [31,55,56,57]. As higher educational attainment is usually associated with better interaction with new media, which in turn may be affected by significant misinformation and false beliefs, this seemly inappropriate and paradoxical association may be at least partially explained. On the contrary, the eventual results of our study could find some clarifications in the very high knowledge status we were able to identify among study participants, which in turn was significantly associated with a better acceptance of the vaccine and a more accurate risk perception of the potential consequences of tetanus natural infection. In this regard, the substantial lack of individuals advocating “personal and/or religious motivations” for avoiding TeV may be linked to the sampling of participants. Previous studies have regularly identified not only people from occupational settings having advocated such barriers as being among the most significant ones but also a considerable share of professional athletes who have advocated “religious exemption” to vaccinations [7,9].

Even though age was not characterized as a main effector for both vaccination status and vaccine hesitancy, in our sample, younger age groups (i.e., <40 y.o. at the time of the survey) not only were characterized by a quite large share of individuals without an appropriate TeV status but also exhibited higher rates of vaccine hesitancy, particularly among amateur players from the age group 20 to 29 y.o. (i.e., 41.5%). Moreover, a clear age-dependent trend was identified, with a decreasing share of vaccine-hesitant responders in older age groups. A possible explanation may be found in the model we applied to define a vaccine hesitancy status, with potential consequences that may exceed the limited scope of this research. A specificity of the TTM is the introduction of the “time” factor, as participants are asked about an action to be taken in a foreseeable future, a variable that is often underestimated [30,35]. A young, healthy individual may reasonably fail to perceive any pressure towards TeV. Even though tetanus was perceived as a severe disease, it is also more difficult to be contracted in usual settings when compared with measles, influenza or even COVID-19. On the contrary, the increasing awareness of official requirements for vaccination against and the potential severity of tetanus infection may lead to improved understanding of the corresponding requirements. Even though forgetfulness about TeV shots may appear to be a somewhat indolent and scarcely important factor, it should be stressed that having forgot a periodic vaccination shot has been identified often among the most frequently reported causes for an inappropriate TeV status [7,13,14] and that all delays may eventually evolve in improper immunization rates, with a subsequent increased risk for developing tetanus when interacting with tetanus spores.

*Limitations*. Our study is affected by some significant limitations. First, even though the preventive sample size calculation suggested that our study may be substantially representative of the targeted population, our estimates should be taken with caution. As we lack appropriate data on the TeV rates in Italian athletes, the sample size was calculated by means of available proxies [7,9,10,11,12,58]. Moreover, as our sample included only 0.06% of the target population, a generalization of our findings is forcibly limited. In this regard, it should be stressed that our questionnaire was designed and shared before the inception of the ongoing SARS-CoV-2 pandemic. As COVID-19 and its vaccination campaign have significantly impacted the acceptance of vaccines in the general population, we cannot rule out that whether the actual KAP of the study recipients is still considered representative of targeted population [59].

Second, being based on an Internet-based questionnaire, our study shares all of the limitations of these innovative instruments [60,61,62]. Despite the substantial reliability, the cost-effectiveness, and the reduced turnaround time, web-based surveys are affected by some degree of the “self-selection” of participants, with potential oversampling of certain subgroups. Due to their better literacy or younger age, some subjects who are more accustomed to sharing personal information through the Internet also exhibit more proactive attitudes or greater knowledge about the topic assessed, eventually impairing the representativity of the original population. Similarly, not participating in the survey could be understood as a negative attitude or a lack of knowledge about the targeted topic [61]. In this regard, our sample was certainly affected by some degree of self-selection, as suggested by the oversampling of subjects from younger age groups and higher educational attainment, but it should be stressed that our target population was represented by active amateur players, and basketball in European countries is far more popular among these specific subgroups of the general population.

Third, because of their design, some of the items assessed through the knowledge test may have been affected by the “social desirability bias”, with participants more frequently reporting “common sense” and “socially appropriate” answers than their actual understanding of the item assessed [63,64]. Therefore, our results could have ultimately overstated the share of individuals with an effective understanding of TeV and tetanus syndrome.

Fourth, as our study had no external validation, we cannot rule out that some of the respondents did not fully adhere to our selection criteria, furtherly compromising the actual representativity of the sample. For the very same reasons, the self-reported vaccination rates should also be taken with caution. Even though the actual consistence of self-reported TeV status with actual immunization status may be quite reliable [65], participants in younger age groups may have failed to recall the vaccination shot in age 12 to 18 years, as TeV was included in a multiple formulate, with a potential overestimation of individuals with inappropriate vaccination status [3,7,25]. Similarly, while asking the participants about their last booster may have improved our capability to identify individuals who specifically received TeV in certain settings (i.e., occupational settings, emergency department, etc.), we cannot rule out that individuals involved in more extensive vaccination strategies with multiple formulates (e.g., women with previous pregnancies) had improperly recalled their status [66,67]. On the contrary, having not inquired about the number of shots that participants actually received and were able to recall, a certain number of individuals that were immunized at an adult age may have improperly identified their basic status as appropriate [68].

Fifth, since our study focuses on Italy, which has specific requirements for TeV, it is neither typical nor representative of all developed countries. As Italian law enforces both the medical surveillance of workers and athletes, with corresponding health services ultimately available to potential recipients, and TeV as being mandatory, our results cannot be easily comparable with other high-income countries, even in European settings, where the common European Union framework should guarantee greater homogeneity [7,9,10,39].

Sixth, our study implemented TTM in a study of vaccine hesitancy. By design, TTM is particularly able to “follow” the individual through the stages of change and may also be useful in assisting the design of intervention strategies effective at moving the person to the next stage of change and subsequently through the model to maintenance [30,33,35]. A cross-sectional design, therefore, may be quite unable to properly catch and follow the progression of the targeted individuals from a stage to another, particularly when the “stages” represent adjacent segments of an underlying continuum. A prospective longitudinal study could enable stronger inferences to be drawn on this specific topic [69].

Lastly, our study shares the limitations of the TTM when it is applied in public health settings. For instance, the TTM usually ignores the social context in which change occurs. By focusing on the inner triggers, our model may fail to address externals pressures moving the participants towards “appropriate” behaviors (i.e., accepting vaccination shots) [29,30,35]. However, it should be stressed that the legal framework of TeV, both in occupational and sports settings, has guaranteed a more homogenous context, minimizing the impacts and the characteristics of the various external pressures. Second, the lines between the TTM stages have been often perceived as arbitrary, with no set criteria of how to determine a person’s stage. This criticism may be minimized when dealing with interventions such as vaccinations, as specific stages (i.e., interaction with a medical professional, booking of the vaccine, receiving the vaccine and noting periodic shots) are quite easier to objectivize compared with the stages of quitting smoking, alcohol drinking, etc. Similarly, as immunizations are somewhat time-dependent (e.g., duration of the effective protection guaranteed by a vaccine; vaccination schedules, etc.), the usual criticism towards understanding the duration of the various stages may be minimized [35]. On the contrary, another usual criticism towards TTM is more difficult to address even in this specific setting: the assumption that individuals make coherent and logical plans in their decision-making processes. In other words, we cannot rule out that individuals that seemly have rejoined a preparation stage or even the action stage (through the booking of a vaccination shot) may in fact fall back. With vaccine acceptance and vaccine hesitancy being quite dynamic processes, even a maintenance status may improperly regress to earlier stages because of unplanned and emotive triggers. In this regard, it is important to stress that vaccines have been often affected by similar events, for example, the claims towards severe side effects of seasonal influenza vaccines, both in Italy and in France; the alleged links between the HBV vaccine and multiple sclerosis [70,71,72]; and the possible association between peri- and myocarditis and thrombosis with SARS-CoV-2. In all of the aforementioned cases, false claims of vaccine safety have negatively contributed to global efforts in improving corresponding vaccination rates [73,74]. This is quite important when dealing with TeV because the fear of side effects is regularly listed among the main barriers for vaccination, as for our study [12,13].

## 5. Conclusions

Our study suggests that Italian amateur BPs exhibit vaccination rates that substantially exceed usual estimates for the general population. Despite the extensive acceptance of TeV, up to a third of respondents exhibited a certain degree of vaccine hesitancy, which was mainly associated with non-modifiable factors such as gender, level of formal education, and occupational background from healthcare settings. However, as vaccine hesitancy was addressed by means of the TTM, which in turn hinted towards a reduced share of vaccine-resistant individuals, interventions focusing on the main barriers reported by the study participants may eventually improve the overall acceptance of TeV. As knowledge status was associated with a more appropriate risk perception, it is reasonable that addressing residual false beliefs and misinformation might improve the attitudes of these relatively young individuals. As tetanus infection may be effectively avoided by TeV, improving the vaccination rates is, therefore, instrumental and cost-effective in reducing the potential burden of such usually deadly syndrome.

## Figures and Tables

**Table 1:** Characteristics of the 270 amateur basketball players participating to the study (Italy, 2020).

	No./270, %	Average ± S.D.
Age (years)		36.9 ± 12.0
Age > 50 years	36, 13.3%	
Gender		
Male	115, 42.6%	
Female	155, 57.4%	
Formal education		
Primary school	6, 2.2%	
Secondary school	103, 38.2%	
University or higher	161, 59.6%	
Migration background	5, 1.9%	
Living with children	85, 31.5%	
Working in healthcare settings or affiliate	93, 34.4%	
Working in settings requiring TeV	35, 13.0%	
Potential exposure to tetanus in hobbies/personal activities	123, 45.6%	
Previous interaction with tetanus case(s)	19, 7.0%	
Appropriate tetanus vaccination status	197, 73.0%	
Last vaccination shot performed by one of the following:		
Personnel of the competent Local Health Unit	128, 47.4%	
General Practitioner	20, 7.4%	
Occupational Physician	9, 3.3%	
Personnel of an Emergency Department	8, 3.0%	
Information not provided	32, 11.9%	
Unable to recall the last vaccination shot	73, 27.0%	
General practitioner checked TeV (ever)	73, 27.0%	
Sport physician checked TeV (ever)	47, 17.4%	
Occupational physician checked TV (ever)	69, 25.6%	
Previously checked by an healthcare provider	100, 37.0%	
Any hesitancy towards TeV	90, 33.3%	
General Knowledge Score (%)		79.8 ± 16.6
General Knowledge Score > median (83.3%)		94, 34.8%
Tetanus is a severe/very severe disease	246, 91.1%	
Tetanus is a common/very common disease	161, 59.6%	
TeV is potentially associated with severe/very severe side effects	13, 1.1%	
TeV is associated with common/very common side effects	39, 6.3%	
Risk Perception Score—natural infection (%)		63.9 ± 26.6
Risk Perception Score—vaccine (%)		14.0 ± 15.4
Risk Perception Score—natural infection > median (60.0%)	114, 42.2%	
Risk Perception Score—vaccine > median (8.0%)	116, 43.0%	

Note: TeV = tetanus vaccine; appropriate tetanus vaccinations status was defined as a complete set of TeV with one booster shot against tetanus within the last 10 years.

**Table 2:** Knowledge test: response distribution of presented items proposed to the 270 amateur basketball players participating in the survey and contributing to the assessment of general knowledge score (GKS) (Cronbach’s alpha = 0.745).

Statement	Correct Answer	No., %
Tetanus may be acquired through improperly managed burns.	True	93, 34.4%
Tetanus may be acquired through injuries contaminated by earth and dusts.	True	244, 90.4%
Additives contained in vaccine formulates may elicit severe health effects.	False	207, 76.7%
Some immunizations may elicit auto-immune diseases.	False	270, 100%
Some vaccines increase the risk for developing allergic disorders.	False	216, 80.0%
Vaccines are nowadays useless; infectious diseases can be treated through specific drugs.	False	239, 88.5%
Without vaccines, smallpox would still exist.	True	254, 94.1%
The efficacy of vaccines has been repetitively proven.	True	257, 95.2%
In Italy, tetanus vaccines are associated with specific legal requirements.	True	167, 61.9%
Children would be more resistant to natural infections if unvaccinated.	False	239, 88.5%
Some vaccinations are administered too early.	False	207, 76.7%
The immune system may be overloaded by the current frequency of vaccines required for school.	False	192, 71.1%
Tetanus vaccine is required for sport activities, even for basketball.	True	82, 30.4%

**Table 3:** Frequency of respondent agreeing with statements about the process of change used in the study.

	Status According the TTM	No., %
I am not interested in obtaining the tetanus vaccine, ever.	Precontemplation	8, 3.0%
I am not interested in obtaining the tetanus vaccine within the next 6 months.	Precontemplation	31, 11.5%
I am uncertain whether I am interested in obtaining the tetanus vaccine.	Contemplation	7, 2.6%
I am considering discussing the tetanus vaccine with a physician.	Contemplation	4, 1.5%
I am interested in obtaining the tetanus vaccine within the next 6 months but have no appointment booked yet.	Contemplation	40, 14.8%
Somewhat hesitant		90, 33.3%
I am interested in obtaining the tetanus vaccine within the next 30 days but have no appointment booked yet.	Preparation	9, 3.3%
I have booked a vaccination appointment.	Action	6, 2.2%
I have received my first vaccination shot but have no plans for further shots.	Action	12, 4.4%
I have received my first vaccination shot; I have noted the need for further shots.	Maintenance	76, 28.1%
I have received my first vaccination shot; I have appointments for further shots.	Maintenance	16, 5.9%
I have completed the vaccination schedule; I have noted the need further shots.	Termination	61, 22.6%
Somewhat Favorable		180, 66.7%

Note: TTM = transtheoretical model.

**Table 4:** Frequency of perceived barriers and motivators towards tetanus vaccination among 270 amateur basketball players participating into the survey.

Barriers	No./73, %
Fear of side effects	46, 63.0%
Doubts on the efficacy/safety of vaccines	12, 16.4%
Doubts about the producers of vaccines	7, 9.6%
Forgot periodic shot	4, 5.5%
Preference of alternative measures	4, 5.5%
Personal motivations, undisclosed	0, -
Religious motivations	0, -
Motivators	No./197
Avoiding tetanus	111, 56.3%
TeV was recommended by professionals at emergency departments after an injury	14, 17.1%
TeV is required by some personal activities	26, 13.2%
TeV is legally required in my workplace	11, 5.6%
TeV was recommended by a GP	9, 4.6%
TeV is required by my employer	3, 1.5%
TeV was recommended by an SP	3, 1.5%
TeV was recommended by an OP	3, 1.5%

Note: GP, general practitioner; SP, sport physician; OP, occupational physician.

**Table 5:** Univariate association of individual characteristics of 270 amateur basketball players participating in the survey and reporting an appropriate vaccination status against tetanus (i.e., having received a full basic immunization course including three separate doses and at least one vaccination shot in the 10 years preceding the study) and with vaccine hesitancy (dichotomized as none vs. any).

	Appropriate TeV Status			Any Vaccine Hesitancy		
	Yes (No./197, %)	No (No./73, %)	*p* Value	Yes (No./90, %)	No (No./180, %)	*p* Value
Male gender	72, 36.5%	43, 58.9%	0.002	52, 57.8%	63, 35.0%	0.001
Aged ≥ 40 y.o.	116, 58.9%	50, 68.5%	0.193	63, 70.0%	103, 57.2%	0.057
Formal education—university or higher	133, 67.5%	28, 38.5%	<0.001	40, 44.4%	121, 67.2%	<0.001
Migration background	3, 1.5%	2, 2.7%	0.880	2, 2.2%	3, 1.7%	1.000
Children in the household	64, 32.5%	21, 28.8%	0.662	33, 36.7%	52, 28.9%	0.247
Previous interactions with tetanus	15, 7.6%	4, 5.5%	0.733	2, 2.2%	17, 9.4%	0.053
Working in healthcare settings	68, 34.5%	25, 34.2%	1.000	20, 22.2%	73, 40.6%	0.004
Occupational requirement for TeV	20, 10.2%	15, 20.5%	0.040	15, 16.7%	20, 11.1%	0.276
Hobbies at risk for tetanus	90, 45.7%	33, 45.2%	1.000	44, 48.9%	79, 43.9%	0.517
TeV checked by SP	23, 11.7%	24, 32.9%	<0.001	25, 27.8%	22, 12.2%	0.003
TeV checked by OP	56, 45.5%	13, 29.5%	0.095	14, 25.9%	55, 48.7%	0.009
TeV checked by GP	57, 28.9%	16, 21.9%	0.318	12, 13.3%	61, 33.9%	0.001
TeV checked, any healthcare provider	77, 39.1%	23, 31.5%	0.316	25, 27.8%	75, 41.7%	0.036
GKS > median value	74, 37.6%	20, 27.4%	0.157	20, 22.2%	74, 41.1%	0.003
RPS-T > median value	92, 46.7%	22, 30.1%	0.021	34, 37.8%	80, 44.4%	0.360
RPS-V > median value	74, 37.6%	42, 57.5%	0.005	45, 50.0%	71, 39.4%	0.128
Vaccine hesitancy (any)	37, 18.8%	53, 72.6%	<0.001	-	-	-
Appropriate TeV status	-	-	-	37, 41.1%	160, 88.9%	<0.001

Notes: GP, general practitioner; SP, sport physician; OP, occupational physician; TeV, tetanus vaccine; GKS, general knowledge score; RPS, risk perception score; RPS-T, RPS for tetanus syndrome; RPS-V, RPS for TeV.

**Table 6:** Multivariable analysis of the association between individual characteristics from 270 amateur basketball players participating in the survey and reporting an appropriate tetanus vaccination status (TeV; having received a full basic immunization course including three separate doses and at least one vaccination shot in the 10 years preceding the study) and with vaccine hesitancy (dichotomized as none vs. any). Adjusted odds ratios (adjOR) were calculated by means of a binary logistic regression analysis that included all factors that, in the univariate analyses, were associated with an appropriate status for TeV and vaccine hesitancy (i.e., *p* < 0.05).

	Appropriate TeV Status		Vaccine Hesitancy	
	adjOR	95%CI	adjOR	95%CI
Male gender	0.527	0.269; 1.032	3.148	1.072; 9.244
Formal education—university of higher	1.784	0.886; 3.592	0.065	0.013; 0.319
Working in healthcare settings	-	-	0.042	0.007; 0.265
Occupational requirement for TeV	0.645	0.235; 1.766	-	-
TeV checked by SP	0.433	0.187; 1.005	3.138	0.661; 14.910
TeV checked by OP	-	-	0.181	0.024; 1.347
TeV checked by GP	-	-	0.126	0.014; 1.126
TeV checked, any healthcare provider	-	-	1.199	0.110; 13.106
GKS > median value	-	-	3.099	0.790; 12.158
RPS-T > median value	1.786	0.870; 3.666	-	-
RPS-V > median value	0.641	0.321; 1.280	-	-
Vaccine hesitancy (any)	0.115	0.059; 0.225	-	-
Appropriate TeV status	-	-	0.030	0.006; 0.151

Note: TeV, tetanus vaccine; SP, sport physician; OP, occupational physician; GP, general practitioner; GKS, general knowledge score; RPS, risk perception score; RPS-V, RPS for vaccination; RPS-T, RPS for natural infection.

## Data Availability

The data presented in this study are available from the corresponding author upon request.

## Appendix A. English Translation of the Questionnaire

V01	Tetanus Basket	Authors’ translation
Estimated reader, We invite you to take a survey on your knowledge about, attitudes toward and practices regarding the tetanus vaccine. Our aim is to identify the main effectors and barriers towards tetanus vaccination to better understand which interventions may be useful and effective in improving vaccination rates in amateur athletes. This survey is completely voluntary. There are no negative consequences if you do not want to take it. If you start the survey, you can always change your mind and stop at any time. All information that is requested are defined in broad and generic terms, and all data will be handled anonymously. After the completion of the survey, there is no way to associate any questionnaire to any individual, as data such as email address or IP address (of the computer, smartphone, tablet, etc.) from the device employed to fill the questionnaire are neither requested nor collected according to the data-protection law. Regarding the individual data included in this questionnaire, we stress that only strictly necessary demographic information (e.g., gender, age, etc.) will be eventually requested, but as previously reported, there is no way to link this information to the individual. According to the Regulation (EU) 2016/679 of the European Parliament and of the Council of 27 April 2016 on the protection of natural persons, we adhere to the following: - All collected data are retrieved only for scientific aims: no information will be shared with third parties without the consent of the individual. However, all individual data can be requested by a legal prosecutor when requested specifically in accordance with the national law, even without personal consent; this is the only exception legally admissible and statutorily requested regarding the aforementioned confidentiality agreement; - After the completion of the questionnaire, it will be impossible to ascertain between individual participants: as the questionnaire will be managed completely anonymously, no modifications, corrections or even removal of data will be possible; - The collected data will be stored only for the time required by this study. - The person responsible for processing personal data is Dr. Riccò Matteo (tel. XXXXXXXX), who will provide further information regarding data management if requested.		

Do you agree to participate in this survey?	YES ( ) NO ( ) end of questionnaire
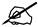

Do you live in Italy?	YES ( ) NO ( ) end of questionnaire
Are you practicing basketball in any amateur division? (In other words, are you practicing basketball in a formally registered basketball team, irrespective of division, geographic area and level of competition? If you play basketball on a regular basis but you are not affiliated with a formally registered basketball team, please answer “no”)	YES ( ) NO ( ) end of questionnaire
Do you benefit from a salary/any economic wage/economic benefits from your team?	YES ( ) NO ( ) end of questionnaire

SECTION 1

Have any of your friends/neighbors/relatives ever been diagnosed with tetanus?	YES ( ) NO ( )
Do you have any of the following occupations or hobbies? [see Appendix B]	YES ( ) NO ( )
Has your employer enforced any requirement or recommendations for tetanus vaccination?	YES ( ) NO ( )
Do you work in healthcare settings?	YES ( ) NO ( )
To the best of your knowledge, have you received a basic vaccination schedule for tetanus *?	YES ( ) NO ( )
To the best of your knowledge, have you received one booster shot against tetanus within the last 10 years (irrespective of its settings and motivations)? **	YES ( ) → go to SECTION 1.a NO ( ) → go to SECTION 1.b
* = A basic vaccination schedule is defined by all of the required shots for newborns plus another dose at age 6 and another dose at ages 12 to 18 or, in adults without previous TeV or with an unknown vaccination status, four doses (T0, T + 4 weeks, T + 6 months and T + 1 year) plus another dose in the following year.	
** = Please check “yes” if you received the booster dose required for individuals aged 12 to 18 years.	
Section 1. Motivators: please report the reasons that motivated you to become vaccinated against tetanus.	
Avoiding tetanus	( )
TeV was recommended by professionals at the emergency departments after an injury	( )
TeV is required by some personal activities	( )
TeV is legally required in my workplace	( )
TeV was recommended by a GP	( )
TeV is required by my employer	( )
TeV was recommended by an SP	( )
TeV was recommended by an OP	( )
Section 2. Barriers: please report the reasons that caused you to refrain from being vaccinated against tetanus.	
Fear of side effects	( )
Doubts on the efficacy/safety of vaccines	( )
Doubts on the producers of vaccines	( )
Forgot periodic shot	( )
Preference of alternative measures	( )
Religious motivations	( )
Other reasons, undisclosed	( )
Last vaccination shot was performed by	Personnel of a competent local health unit ( ) General practitioner ( ) Occupational physician ( ) Personnel of an emergency department ( ) Unwilling to respond ( ) Unable to recall the last vaccination shot ( )
In recent years, have any of the following medical professionals ever checked your tetanus vaccination status?	General practitioner ( ) Sport physicians ( ) Occupational physician ( ) Any other healthcare provider ( )
According to your understanding and regarding its diffusion in the general population, tetanus is a disease.	1—Not significant (of no significant concern in daily practice) 2—Slightly significant 3—Somewhat significant 4—Moderately significant 5—Very significant (of very high concern in your daily practice)
According to your understanding and regarding its severity in the Italian working population, tetanus is a disease.	1—Not significant (of no significant concern in daily practice) 2—Slightly significant 3—Somewhat significant 4—Moderately significant 5—Very significant (of very high concern in your daily practice)
According to your understanding, tetanus vaccination is potentially associated with side effects that are (frequency)	1—Not significant (of no significant concern in daily practice) 2—Slightly significant 3—Somewhat significant 4—Moderately significant 5—Very significant (of very high concern in your daily practice)
According to your understanding, tetanus vaccination is potentially associated with side effects that are (severity)	1—Not significant (of no significant concern in daily practice) 2—Slightly significant 3—Somewhat significant 4—Moderately significant 5—Very significant (of very high concern in your daily practice)

SECTION 2. In the following section, a series of statement will be provided. Some are true; some are false. Please mark the following statement according to your current understanding.

	True	False	Do Not Know
Tetanus may be acquired through improperly managed burns.	[ ]	[ ]	[ ]
Tetanus may be acquired through injuries contaminated by earth and dusts.	[ ]	[ ]	[ ]
Additives contained in vaccine formulates may elicit severe health effects.	[ ]	[ ]	[ ]
Some immunizations may elicit auto-immune diseases.	[ ]	[ ]	[ ]
Some vaccines increase the risk for developing allergic disorders.	[ ]	[ ]	[ ]
Vaccines are nowadays useless; infectious diseases can be treated through specific drugs.	[ ]	[ ]	[ ]
Without vaccines, smallpox would still exist.	[ ]	[ ]	[ ]
The efficacy of vaccines has been repetitively proven.	[ ]	[ ]	[ ]
In Italy, tetanus vaccines are associated with specific legal requirements.	[ ]	[ ]	[ ]
Children would be more resistant to natural infections if unvaccinated.	[ ]	[ ]	[ ]
Some vaccinations are administered too early.	[ ]	[ ]	[ ]
The immune system may be overloaded by current frequency of vaccines required for school.	[ ]	[ ]	[ ]
Tetanus vaccine is required for sport activities, even for basketball.	[ ]	[ ]	[ ]

SECTION 3. Please mark the statement that most precisely reflects your status regarding tetanus vaccination.

I am not interested in obtaining the tetanus vaccine, ever.	( )
I am not interested in obtaining the tetanus vaccine with the next 6 months.	( )
I am uncertain whether I am interested in obtaining the tetanus vaccine.	( )
I am considering discussing tetanus vaccine with a physician.	( )
I am interested in obtaining the tetanus vaccine within the next 6 months but have no appointment booked yet.	( )
I am interested in obtaining the tetanus vaccine within the next 30 days but have no appointment booked yet.	( )
I have booked a vaccination appointment.	( )
I have received my first vaccination shot but have no plans for further shots.	( )
I have received my first vaccination shot; I have noted the need for further shots.	( )
I have received first vaccination shot; I have made appointments for further shots.	( )
I’ve completed the vaccination schedule, I have noted the need for further shots	( )

SECTION 4. Characteristics of the compiler.

Eventually, we will request some personal information from you.	
You are (by your personal understanding)	
Male	( )
Female	( )
Rather not answer	( )
Year of birth	___________
Your highest achievement in terms of education:	( ) Primary school (<8 years of formal education) ( ) Secondary school (8–13 years of formal education) ( ) University or higher
Were you or any of your parents born abroad?	YES ( ) NO ( )
Do you live with subjects aged 12 years or less?	YES ( ) NO ( )

## Appendix B

Occupations and occupational settings where tetanus vaccination mandate has been enforced by Law No. 292/1963, article 2.

- Farmers and agricultural workers;
- Shepherds;
- Cattle breeders;
- Hostlers;
- Horse riders;
- Tanners;
- Janitors and personnel managing hippodromes;
- Scavengers/(street) sweepers;
- Road maintenance workers;
- Diggers/laborers;
- Miners;
- Kilnsmen;
- Construction workers;
- All railway workers;
- Ragmen;
- Garbage collectors/people managing wastewater;
- Workers from the industry of paper and cardboard;
- Carpenters and joiners;
- Workers from metallurgical industries.

## Appendix C. List of Acronyms

BP	basketball player
GKS	general knowledge score
GP	general practitioner
OP	occupational physician
RPS	risk perception score
RPS-T	risk perception score, tetanus
RPS-V	risk perception score, vaccine
SP	sport physician
TeV	tetanus vaccine
TTM	transtheoretical model
